# Cases of Obscure Disease of the Lung

**Published:** 1854-05

**Authors:** F. W. Lewis


### MEDICAL EXAMINER.

NEW SERIES.—NO. CXIII.—MAY, 1854.

### ORIGINAL COMMUNICATIONS.

### *Cases of obscure Disease of the Lung.*

By F. W. Lewis, M. D.

The peculiar interest which attaches to the diagnosis of pulmonary diseases, together with the obscurity in which the subject is still involved, even with all the aids furnished by modern science, have induced the writer to report the following cases, all of which came under the observation of distinguished practitioners. Although the subjoined cases might be deemed examples of chronic pneumonia, a disease occasionally met with among the poorer classes in this city, and as such hardly meriting to be brought to the notice of the profession, yet he thinks that on a close examination they will be found to present complications novel and unusual in that affection; and which, in the one case preceding and the other following it, served to mask the symptoms, and render a correct diagnosis very difficult if not impossible.

Case 1.—Martha Moore, æt. 7, (Twelfth street above South,) of scrofulous habit and antecedents, was attacked about the middle of September, 1851, with symptoms of pleurisy, which from neglect and want of proper medical advice terminated in a pleuropneumonia of the right side. When first seen by me, on the 4th of October, she presented the following symptoms:—considerable emaciation, sallow semi-cyanosed tint, short dry cough, intense dyspnœa, with constant tendency to syncope. The prostration was extreme; pulse averaged 135 per minute, very feeble and irritable; respirations 45. The physical signs consisted in absolute dulness on percussion over the right side of the thorax, both anteriorly and posteriorly; very feeble respiration at the apex of the lung, passing beneath the spine of the scapula into a coarse crepitation, below this point total absence of respiratory murmur; tubal breathing in the right interscapular space. The left lung afforded no indications of disease. The heart was in its normal position and the sounds were healthy. The patient remained in much the same condition up to the 23d, daily losing flesh, and suffering from most distressing difficulty of breathing, amounting during most of the time to orthopnœa. Occasionally she expectorated small quantities of exceedingly tough mucus, tinged with blood, and not unfrequently during the paroxysms of cough syncope would supervene. About this date respiration ceased to be heard at any point of the right lung, and the dulness on percussion was absolute. A muco-serous diarrhœa set in, and the patient had repeated and exhausting hemorrhages from the nose. By the first of November emaciation had progressed to a painful degree. She had constant hectic and profuse night sweats. The orthopnœa continued urgent, and from a constant habit of sitting propped up in bed, considerable lateral deviation of the spine to the right had taken place. Soon after this a peculiar and unnatural prominence of the anterior and lower part of the right thorax was noticed, the intercostal spaces retaining their normal appearance. But little attention was at first paid to this phenomenon, which continued to increase, until by the latter part of November, the actual difference by measurement between the right and left side, at the base of the thorax, amounted to two inches and a half, causing a deformity the more remarkable from the apparent contraction of the upper portion of the chest. By the middle of December this swelling had attained considerable dimensions, projecting forwards, outwards, and downwards, and presenting an abrupt upheaval of the parietes of the chest from about the third rib down to the base.

At this date (January 5th) the disease appeared to have reached its acme, the difference between the two sides being over three inches. The patient had wasted nearly to a skeleton, had profuse night sweats and exhausting diarrhœa, coughed incessantly, occasionally expectorating blood in small quantities. The physical signs were modified by the appearance of a mucous râle under the clavicle and posteriorly at the base of the lung—but otherwise no change was noted.

About the middle of January the patient began to improve. The color and appetite returned in a measure. The dyspnœa became less urgent. Gradually, as convalescence set in, the diarhœa, hectic and night sweats disappeared. Respiration became audible at the posterior inferior part of the lung, but not anteriorly, slowly extending upwards without the supervention of râles of any kind. It was not before the 1st of May that the health of the patient was completely restored, and the lung had resumed its full functions. The prominence of the anterior wall of the chest, though much diminished, was still obvious, and returned a dull sound on percussion, and it was at least two months later before all traces of the local disease had disappeared.

The treatment of the case throughout was essentially tonic, being mainly directed to sustaining the vital forces of the patient. Counter-irritation was resorted to in various forms. It was while under the use of cod liver oil and a strong ointment of iodine, advised by Dr. W. E. Pepper, (to whom the writer is indebted for many valuable suggestions in connection with the diagnosis and treatment of the case,) that the first symptoms of amendment began to manifest themselves. The subnitrate of bismuth proved particularly valuable in moderating and finally arresting the diarrhœa when all other remedies had failed.

The most interesting feature of the above case is the strong probability of the existence of a large scrofulous tumor at the anterior and lower part of the right thoracic cavity. In hastily reviewing the symptoms, there are two points which seem more specially to support this view, viz.: the peculiar circumscribed prominence of the walls of the thorax at the anterior and lower part, and the non-obliteration of the intercostal spaces; and these taken in connection with the history of the physical signs during the progress of the case, leave but little room for doubt. To any one who has observed the comparative frequency of thickening and deposit of false membranes in the pleura pulmonalis of children after attacks of pneumonia, (more especially when a scrofulous diathesis prevails,) a thickening sometimes amounting to nearly half an inch, it will not appear remarkable that the constitutional tendency should further modify these morbid products even to the development of such a tumor as the above. The final recovery of the patient after such a protracted and apparently hopeless complication of diseases is also a point of great interest.

CASE 2. Mary Tiernay, æt. 35, seamstress, of delicate constitution and nervous temperament, in the month of December, 1852, had an attack of acute articular rheumatism. During the course of this the heart became implicated. This affection was at the time supposed to be endocardial, being characterized by considerable disturbance of the respiration, a livid hue of face, irregular and tumultuous action of the head, distant souffle and great prostration. It was treated by blistering, cupping and mercurials. The acute symptoms soon declined, and by the first week of January she had regained her usual health, complaining only of slight oppression and weight over the precordial region, and frequent attacks of palpitation after active exertion. At this time auscultation revealed a rough double bellows murmur over the sternum, disappearing towards the apex of the heart. This was referred to the aortic valves, but from the great turgescence of the veins of the neck and face, the livid tint and good radial pulse it was thought not improbable that there might be a coexisting disease of the valves of the pulmonary artery. During the ensuing month she was several times examined and the same phenomenon always found present. Dr. F. W. Sargent, by whom the case was seen, also discovered that the respiration over the right side was relatively feebler than over the left, but on questioning the patient it did not appear that she had ever suffered from an affection of the corresponding lung.

With these exceptions, nothing to cause uneasiness was noticed up to the morning of the 2d of February, 1853, when, on going up a flight of stairs, the patient was suddenly seized with intense dyspnœa, accompanied by an insupportable feeling of tightness and oppression over the heart and a deadly faintness. During the day she continued in a very critical condition, having several alarming attacks of syncope. Next day, when I saw her, she presented the following symptoms: Livid, cyanosed tint; cool, moist skin; sunken eyes; orthopnœa; short, dry, frequent cough; hurried and irregular respiration, with an exceedingly feeble pulse, about 125 per minute; complained of deep seated sense of uneasiness over the right side; great precordial oppression and extreme weakness. On examination, the right side of the chest was found not to expand with inspiration, and returned a relatively flat sound on percussion. By ausculting, very little if any respiration could be heard over the same side, while on the left, the breathing was exaggerated and almost bronchial throughout. On applying the ear over the heart, its sounds were found tumultuous and irregular, but perfectly sharp and clear; *all traces of the pre-existing bellows murmur had disappeared.*

The symptoms were met by cupping, sinapisms, and nervous stimuli. A large blister was subsequently applied over the right side, without, however, any amendment resulting. For a week the orthopnea and prostration remained stationary, perhaps rather increased; the dulness on percussion also became more decided, and respiration ceased to be heard in the lung, except in the subclavicular space, where a feeble murmur was audible.

A month nearly had elapsed before the system had sufficiently reacted to accommodate itself to the profound lesion of the respiratory apparatus; and during this period the patient was never able to lie upon her back.

About the middle of March a change for the better became perceptible, the strength began to return, and the dyspnœa ceased to torment her to the same extent; but the local lesion as evidenced by the physical signs, underwent no modification. From that date up to the present, a period of over 13 months, the lung has remained in much the same condition, presenting the same absolute dulness and nearly total absence of all respiration, except under the clavicle. The patient's health is feeble and precarious, but she is able to attend to her household work without material inconvenience.

It is not without some hesitation, and a consciousness that in the absence of all direct proof such an hypothesis can possess merely a speculative interest, that the writer ventures to advance the opinion that this was probably an instance of consolidation or collapse of the lung, resulting from obstruction and partial obliteration of the right pulmonary artery by an organized coagulum, or other morbid deposit, detached from the right side of the heart, and washed by the current of circulation into that vessel. Of the possibility of such an accident there can hardly be a question; and a careful investigation of the history of the case, its symptoms and physical signs, will, he thinks, if not prove, at least show the probability of its occurrence.

In briefly reviewing the leading points of the case, we find the primary affection to have been rheumatic endocarditis, ending in deposit of lymph within the ventricles, and giving rise to a rough bellows murmur indicative of some valvular affection; not, however, materially interfering with the circulation or protracting convalescence. The lungs at this time are healthy, with the exception of slightly feeble respiratory murmur on the right side, a circumstance giving rise to no suspicion of disease there. A month nearly has elapsed, allowing time for the organization of the morbid inflammatory product, and the patient has regained her customary health, when she is suddenly attacked with the most alarming dyspnœa, and at the same time with signs of profound lesion of the respiratory organs, while the abnormal bruit, until then distinctly audible, finally disappears.

From these symptoms it seems not unreasonable to infer the detachment of a coagulum or morbid growth from the valves, which till then it had partially obstructed.

Almost immediately a suspension of the function of the right lung is observed, and in less than a week's time nearly complete consolidation, or some condition analogous, has taken place, from which the lung has never recovered.

Admitting the probability of such a lesion as the above, an interesting question now suggests itself, as to what effect would most likely be produced by the presence of a firm coagulum, or any consistent organized product washed into the right pulmonary artery and causing an almost total obliteration of its calibre. Would pneumonia, pulmonary apoplexy, effusion or collapse be most likely to result?

In the admirable series of cases of obstruction of the pulmonary artery reported by M. Paget, (Medico-Chirurgical Transactions,) the primary cause of this lesion appears to have been an obstruction of the capillary circulation in a portion of the lung, a consequence of some co-existing disease of the left side of the heart, pneumonia, pulmonary apoplexy, or, finally, from some disease of the artery itself, productive of morbid fatty growths, deficient activity of circulation, or too great plasticity of the blood forming its coagulation. In other respects these cases present points of difference so essential as to render any reasoning from analogy impossible. Of course all speculations on such a question must necessarily be vague and unsatisfactory; yet the idea may be hazarded that all the known conditions present were favorable to the production of collapse of a large portion of the lung. The violent shock to the brain and nervous system reacting on the nerve force of the lung, together with the suddenly-arrested circulation through the pulmonary artery and its branches, in the absence of any coincident affection of the left side of the heart and of the pulmonary capillaries, or loss of contractile power in these last, must necessarily have reduced the amount of blood in the vascular network of the air-cells to a very inconsiderable quantity. Now, inasmuch as the walls of the pulmonary vesicles depend in a great degree for their proper tension and support on the capillaries, collapse of the lung, more or less complete, might not unreasonably be expected to result from the state of partial vacuum in these vessels, above suggested.

In the following case is described a condition of the lung somewhat similar, but originating in an entirely different cause.

Case 3.—Michael Welsh, æt. 24, laborer, was admitted into the Pennsylvania Hospital on the 26th of December, 1849, having on the previous day, while intoxicated, fallen from a bridge, a distance of twenty feet, on the ice, striking upon his head. Previous to the accident his health had been good. On the evening of the day of his admission, symptoms of cerebral inflammation set in, and next morning the extreme lividity of face, labored, suspirious breathing, and a short, dry cough, excited attention to the condition of the respiratory organs. At this time the respiratory murmur over the right side of the chest was found very feeble, and there was slight relative dulness on percussion over the same side. The same day the patient became comatose. The pulse was now observed to be exceedingly slow—55 per minute—and respiration very labored, averaging certainly not more than 8 or 10 per minute. By the evening of the next day little or no respiratory sound was audible in the right side, and the dulness on percussion was very marked. At the same time no r le could be detected at any point of the lung, nor did the breathing appear at all bronchial or exaggerated on the opposite side. No change occurred up to the first of January, six days after admission, when the patient sunk exhausted.

On opening the head, a fracture involving the right temporal bone, and extending obliquely through the petrous portion into the carotid foramen was found. In the right foramen lacerum existed a clot about the size of a walnut, which compressed the pneumogastric, extending for some distance along its sheath. Around this coagulum were traces of inflammatory action, particularly about the point of origin of the posterior nervis.

Unfortunately no examination was made of the lung.

This observation is satisfactory, as it clearly shows that under certain circumstances of disease, a lesion of the par vagum of one side may give rise to alteration, if not suspension of the functions of the corresponding lung. This view was maintained by Wilson, Philip, Magendie and Swan, who found, as the result of experiments on inferior animals, that pneumonia, œdema or paralysis invariably followed section of one of the vagi.

A later observer, Dr. John Reid, arrived at directly opposite conclusions, after a very elaborate and carefully conducted series of observations. In summing up, he expresses his conviction "that a lesion of one of the pneumogastrics does not necessarily or even generally induce disease of the lung on that side." The experiments of these distinguished physiologists were mainly confined to section of the nerve in the lower animals, the organization of whose nervous system is proportionately less delicate, and whose recuperative power is much greater than in man, and moreover, in whom there existed no complication of disease as in the above case.

